# Reliability and validity of the International FItness Scale (IFIS): A systematic review and meta-analysis

**DOI:** 10.1016/j.jesf.2026.200501

**Published:** 2026-07-28

**Authors:** Ran Bao, Yanjie Zhang, Francisco B. Ortega, Clemens Drenowatz, Kai Zhang, Sitong Chen

**Affiliations:** a College of Physical Education, Shanghai University, Shanghai, China; b School of Humanities and Social Science, The Chinese University of Hong Kong-Shenzhen, Shenzhen, China; c Department of Physical Education and Sports, Faculty of Sport Sciences, Sport and Health University Research Institute (iMUDS), University of Granada, Granada, Spain; d CIBER de Fisiopatología de la Obesidad y Nutrición (CIBEROBN), Instituto de Salud Carlos III, Granada, Spain; e Division of Sport, Physical Activity and Health, University of Education Upper Austria, Austria; f Healthy Activity Living and Obesity (HALO) Research Group, Children's Hospital of Eastern Ontario Research Institute, Ottawa, ON, K1H 8L1, Canada; g School of Human Kinetics, Faculty of Health Sciences, University of Ottawa, Ottawa, ON, K1N 6N5, Canada; h The Shenzhen Humanities & Social Sciences Key Research Bases of the Center for Mental Health, Shenzhen University, Shenzhen, 518060, China

**Keywords:** IFIS, Reliability, Validity, Systematic review, Meta-analysis

## Abstract

**Background:**

Reliable and valid physical fitness instruments are necessary for population-level surveillance and monitoring. However, lab-based and field-based measures are often impractical in large-scale studies. Self-rated fitness scales such as the International Fitness Scale (IFIS) offer a feasible alternative. Therefore, the aim of this review was to quantitatively synthesize the available evidence on the reliability and validity of the IFIS.

**Method:**

Four online databases (Ovid MEDLINE, SPORTDiscus, EMBASE and PubMed) were searched for papers published prior was performed August 2025. Studies meeting the following criteria were included: (1) published in English, (2) investigated the validity or reliability of the IFIS, (3) no limitation for population subgroups of the included studies. The risk of bias was assessed through the latest version of the COSMIN (Consensus-based Standards for the selection of health Measurement Instruments) Risk of Bias checklist. The random effects model was employed to compute the pooled effect sizes, the heterogeneity of the average effect size was assessed using I^2^ statistic and Q statistics.

**Results:**

After title and abstract screening, 20 records were assessed for full-text eligibility. Of these, 13 eligible studies (9634 participants) were included in this study. Reliability was assessed in 10 studies, and construct validity was assessed in a total of 11 studies (3 assessed the criterion validity). A substantial test-retest reliability was observed for overall fitness (pooled weighted kappa = 0.750, 95%CI = 0.600-0.848, *Q* = 899.283, *I*^*2*^ = 98.777), cardiorespiratory fitness (pooled weighted kappa = 0.752, 95%CI = 0.612-0.846, *Q* = 816.758, *I*^*2*^ = 98.653%), muscular fitness (pooled weighted kappa = 0.724, 95%CI = 0.630-0.798, *Q* = 350.584, *I*^*2*^ = 96.862%), speed-agility (pooled weighted kappa = 0.764, 95%CI = 0.645-0.847, *Q* = 655.508, *I*^*2*^ = 98.322%), and flexibility (pooled weighted kappa = 0.725, 95%CI = 0.633-0.796, *Q* = 335.094, *I*^*2*^ = 96.717%). The 11studies assessing the construct validity of the IFIS reported that participants who self-rated higher fitness with IFIS had higher objectively measured fitness, but due to different methods used, these results could not be meta-analyzed.

**Conclusions:**

This study shows that the IFIS is a reliable instrument to assess physical fitness in children and adolescents. Acceptable validity was reported across studies, yet could not be meta-analyzed due to diversity in methodologies. More studies are needed to discern its cultural adaptation among different population groups.

## 1 Introduction

Physical fitness (PF), comprising health-related and skill-related fitness, is defined as the ability of your body systems to work together efficiently to allow you to be healthy and perform activities of daily living.^1^ PF is a well-established marker of health across the lifespan. In youth, higher levels of cardiorespiratory fitness (CRF) and muscular fitness (MF) are associated with physiological outcomes, including lower adiposity and reduced cardiometabolic and vascular risk, as enhanced psychological well-being and cognitive function in later life.^2^, ^3^, ^4^ Notably, CRF has been shown to be a strong predictor of morbidity (e.g., depression, type 2 diabetes), all-cause mortality (e.g., cardiometabolic and vascular diseases, cancer) and mental illness among adults.^5^ Therefore, previous studies unanimously reinforced the need to test PF in public health monitoring systems,^6^ and the World Health Organization (WHO) also advocates that promoting PF should be targeted as a public health priority.^7^ Notably, several studies revealed that PF levels witnessed declines among youth^8^ and adults^9^ in recent years. A longitudinal study among German adults, further found that PF levels declined with increasing age.^10^ Scientific evidence also showed that PF levels of Chinese children and adolescents continued to drop over the past two decades after 1995,^11^ and a similar finding was found in Chinese adults.^12^

Although PF can be objectively measured and assessed by laboratory or field-based fitness tests in clinical settings,^13^ and school settings,^14^ it might not be feasible in large-scale epidemiologic studies^15^ and in other settings in which there is not enough time, space or equipment for fitness testing. As an alternative, self-rated fitness scales have been posited as a viable alternative for the assessment of PF.^15^ Several existing fitness scales have been developed, including the Physical Self-perception Profile (PSPP),^16^ or the Self-Reported Fitness (SRFit).^17^ However, these instruments are often designed for specific populations and may have limited applicability across age groups.^18^ To address this limitation, Ortega et al. developed the International Fitness Scale (IFIS) as a cost-effective and time-efficient tool as self-report or proxy-report instrument for population-based studies.^19^ The IFIS uses a five-point Likert scale (very poor, poor, average, good and very good) to assess overall PF along with the components CRF, MF, speed-agility (SP-AG), and flexibility (FL).^15^ Since its development, the IFIS has been translated into multiple languages and evaluated across diverse populations and cultural settings.^20^ Furthermore, studies have demonstrated its feasibility for use in large-scale surveillance and population-based research when objective measures are not practical.^21^, ^22^, ^23^ Compared with other self-rated fitness instruments, such as PSPP and SRFit, the IFIS is more concise, applicable across a broader age range, and more suitable for cross-cultural use.

Reliability and validity of an instrument for research use largely determine data quality, statistical analysis and evidence evaluation. Particularly in self-administered study designs, lower reliability and validity of the measurement tool may distort the accuracy of the data and increase the probability of type II error.^24^ Reliability can be evaluated by internal consistency, test-retest stability, and inter-rater reliability.^25^ Validity can be evaluated by content validity (logical validity), criterion validity, and construct validity.^24^ Therefore, before being used in large-scale investigations, newly developed instruments should be evaluated for reliability and validity in different settings.

To our knowledge, only one systematic review has examined the reliability of IFIS.^26^ The pooled reliability estimates in that review were derived from mixed age groups, without accounting for developmental differences in cognitive and psychological maturity across age groups. This may limit the interpretation of the reliability and validity within specific populations. Further, the available validity evidence has not been synthesized systematically. Since the previous review, four reliability studies and six validity studies have been published.^21^^,^^22^^,^^27^, ^28^, ^29^, ^30^ In this context, there is a need for an updated review to synthesize the existing evidence about reliability of IFIS and the first systematic review on validity of IFIS across specific age groups. Therefore, this review sought to synthesize the existing literature and comprehensively assess the reliability and validity of the IFIS.

## 2 Methods

### 2.1 Literature research

This systematic review and meta-analysis follows the 2020 Preferred Reporting Items for Systematic Review and Meta-Analyses (PRISMA) statement (see attached checklist)^31^ and was prospectively registered with INPLASY (202310093). A comprehensive literature search was conducted up to 28 August 2025, using Ovid MEDLINE, SPORTDiscus, EMBASE and PubMed online databases. Before the literature searches, the details of the search strategy were discussed and confirmed by the reviewers. The keywords were: (“International Fitness Scale” OR IFIS) AND (“valid* OR reliab* OR repeatab* OR reproducib* OR accura* OR measurement propert* OR consistenc* OR feasib* OR agreement OR precision”). Additional relevant articles were included via a literature search of the reference lists of the included articles.

### 2.2 Study eligibility criteria

Two reviewers (RB and YZ) separately screened the records, and disagreements between the two reviewers were resolved by a third reviewer (SC). Eligibility criteria were defined as follows: studies were included if they (1) were published in English; (2) examined the reliability and/or validity of the IFIS; and (3) involved any population group without restriction on age, sex, or health condition. No restrictions were applied regarding study population, study design, as long as sufficient data on psychometric properties of the IFIS were reported.

### 2.3 Study appraisal methods

The risk of bias of the included studies was assessed through the latest version of the COSMIN (Consensus-based Standards for the selection of health Measurement Instruments) Risk of Bias checklist, which was developed to improve the credibility of selected health measurement instruments.^32^ According to the “COSMIN methodology for systematic reviews of PROMs—user manual”,^33^ box 6 (reliability), box 8 (criterion validity), and box 9a (construct validity) were used to evaluate the methodological quality of the included studies in this review. The items were rated as inadequate, doubtful, adequate, very good. Furthermore, four GRADE factors were considered for the quality level (including high, moderate, low, and very low) of the included studies, including the risk of bias (i.e. the methodological quality of the studies), inconsistency (i.e. unexplained inconsistency of results across studies), imprecision (i.e. sample size below 100, or 50), and indirectness (i.e. evidence from different populations than the population of interest in the review).^33^ Two reviewers (RB and SC) separately evaluated the quality of the included studies, and disagreements between the two reviewers were discussed with and resolved by a third reviewer (YZ).

### 2.4 Data extraction

The following attributes of the included studies were extracted and synthesized; general characteristics, including the authors of the study, country, target population, sample size, study design, method(s) of measurement, statistical methods, and main findings(s) (Table 1). Moreover, measurement properties, including coefficients of reliability and validity (95% confidence interval). Agreement measures using Cohen's weighted kappa (κ) were reported in the reliability and validity studies. The kappa coefficients were interpreted following the Landis and Koch^34^ benchmarks (Table 4), 0–0.20 = poor coefficients, 0.21-0.40 = fair coefficients, 0.41-0.60 = moderate/acceptable coefficients, 0.61-0.80 = substantial coefficients, 0.81-1.0 = almost perfect coefficients. Notably, construct validity was reported in most of the included studies and the qualitative results of validity were extracted from the included studies.

**Table 1:** Summary and General Characteristics of findings in the Included Studies.

Author/Year	Country	Study population (Age)	Sample size (% girls/female)	Study design	Objective measures				Statistical analysis	Main result(s)
					CRF	MF	SP-AG	FL		
Débora de Almeida et al., 2025 30	Brazil	Adolescents (12-17 years)	Total:319; validity:309; reliability:52	Construct validity, Test–retest reliability (2 weeks apart)	no	no	no	no	① Construct validity: EFA ② Reliability: kappa statistic (kappa coefficients)	① Validity: factor loadings (Factor 1) ranging from 0.46 (Item 5) to 0.85 (Item 1). ② Reliability: weighted Kappa values for overall fitness, CRF, muscle fitness, speed and agility and flexibility were 0.76, 0.80, 0.73, 0.63 and 0.89.
Matelot et al., 2024 22	France (Auvergne Rhone Alpes, Bretagne, Grand Est, Hauts-de-France, Normandie and Pays de la Loire)	Children (10.6 ± 0.9 years)	Validity:2060 (48.8%); reliability: 366 (52.2%)	Construct validity, Test–retest reliability (seven days apart)	20-m shuttle run−walk test (6 min)	the standing broad jump test (SBJ)	running as fast as possible in 5s	the stand-and-reach test	① Construct validity: ANOVA and ANCOVA, *post–hoc* group comparisons ② Reliability: kappa statistic (kappa coefficients)	① Validity: higher self-rated fitness had higher objectively measured fitness. ② Reliability: Test−retest reliability for overall fitness, CRF, muscular strength, speed/agility, and flexibility were 0.65, 0.66, 0.64, 0.59, and 0.72.
Sánchez-López et al., 2023 21	Spain (Granada)	Preschoolers (3–5 years).	Total:3051 (47.4%); reliability: 76	Criterion validity, Convergent validity, Test–retest reliability (2 weeks apart)	20-m shuttle run test	① the handgrip test (upper-body), ②the standing broad jump test (SBJ)	the 4 × 10-m shuttle run test	no	① Validity: ANOVA ② Reliability: kappa statistic (kappa coefficients)	Reliability: the average weighted Kappa was 0.56. Convergent validity: the mean scores of abdominal adiposity variables were significantly higher (p < 0.05) in those with lower parent-reported fitness. Criterion validity: The weighted kappa for the concordance between parent-reported and objective assessment was poor kappa = 0.08-0.18.
Bao et al., 2022 29	China (Shanghai, Nanjing, Wuxi)	Children and Adolescents (13.7 ± 1.5 years)	934 (47.4%)	Test–retest reliability (2 weeks apart)	no	no	no	no	Reliability: kappa statistic (kappa coefficients)	The test–retest weighted kappa coefficients for overall fitness, cardiorespiratory fitness, muscle fitness, speed and agility and flexibility were 0.52, 0.51, 0.60, 0.55 and 0.55.
Henström et al., 2021 28	Sweden (Östergötland)	Pregnant women (31 ± 4 years)	303 (100%)	Construct validity	the 6-min walk test (6MWT)	the handgrip strength test	no	no	ANOVA	Validity: higher self-rated fitness had higher objectively measured fitness.
Romero-Gallardo et al., 2020 27	Spain (Granada)	Pregnant women (32.7 years)	159 (100%)	Construct validity	Bruce test (M = 23.1 ml/kg/min)	the handgrip test (upper-body) (M = 27.6 kg)	no	the back-scratch test (M = 8.4 cm)	① ANOVA ② *post–hoc* group comparisons.	Higher self-rated fitness had higher objectively measured fitness.
De Moraes et al., 2019 35	Brazil (Teresina, Piauí)	Children (3-10 years) Adolescents (11-17 years)	Total: 300 (C: 190; A:110): reliability:149,validity: 131	Criterion validity, construct validity, Test-retest reliability (one week)	20-m shuttle run test	① the handgrip test (upper-body), ②the standing broad jump test (SBJ)	the 4 × 10-m shuttle run test	the sit-and-reach test	① Reliability: kappa statistic (kappa coefficients), ② criterion validity: the agreement, construct validity: kappa concordance coefficient ③ Kruskal–Wallis ANOVA	① Reliability: perfect score (kappa ≥0.93 in children; kappa ≥0.88 in adolescents), ② Validity: moderate criterion validity (kappa ≥0.40 in children and adolescents), moderate construct validity (kappa ≥0.40) in overall fitness, CRF, MF, SP-AG in children and adolescents.
Ramírez-Vélez et al., 2017 36	Colombia (Bogotá)	Youths (9-17.9 years)	Reliability/Validity 229 (46%)	Construct validity, Test-retest reliability (one week)	20-min shuttle run test	① the handgrip test (upper-body), ②the standing broad jump (SBJ)	the 4 × 10-m shuttle run test	the sit-and-reach test	① Reliability: kappa statistic (kappa coefficients), ② Construct validity: ANCOVA and pairwise *post-hoc* (Bonferroni correction).	① Reliability: average weighted kappa of 0.811 ② Construct validity: higher self-rated fitness had higher objectively measured fitness.
Merellano-Navarro et al., 2017 18	Chile (Maule)	Older adults (71.9 ± 7.2 years)	Total: 406 (73%)	Construct validity	6 min aerobic endurance walking test (M = 395.15 m)	① the handgrip test (upper body), ②the 30-s chair stand (lower limb strength)	Timed up and go tests (M = 8.55s)	① Chair Sit-and-Reach Test (lower limbs) (M = −6.70 cm), ② the Back-scratch test (upper limbs) (M = −20.71 cm)	Validity:ANCOVA and *post hoc* comparisons (Bonferroni correction)	① ANCOVA: significant for all physical tests at level p < 0.001, ② *Post-hoc* analysis: significant differences between all categories.
Álvarez-Gallardo et al., 2016 13	Spain (Andalusia)	Women (Fibromyalgia) = 52.3 years; (Control) = 51.3 years	Reliability: 101(fibromyalgia), Validity: 413(fibromyalgia)+195 (control)	Construct validity, Test-retest reliability (one week)	6-min walk test	① the arm curl test (upper body), ②30-s chair stand test (lower body)	the 8-foot up-and-go test	the chair sit-and-reach test	① Reliability (weighted kappa): kappa statistic, ② Construct validity: (ANOVA) and *post hoc* group comparisons (Turkey's correction)	① Reliability: average weighted kappa = 0.45 ② Validity: women with fibromyalgia reported average fitness had better-measured fitness than very poor fitness, control women: higher self-rated fitness had higher objectively measured fitness.
Sánchez-López et al., 2015 37	Spain (Cuenca)	Children (9-12 years)	Reliability/Validity: 245 (54%)	Construct validity test, Test-retest reliability (two weeks interval)	The20-m shuttle run test	① the handgrip test: maximum handgrip strength assessment, ② the standing broad jump test (SBJ)	the 4 × 10-m shuttle run test	the sit-and-reach test	① Reliability(weighted kappa coefficients): kappa statistic, ② Construct validity: ANCOVA	Weighted kappa ranged from 0.64 (FL) to 0.80 (SP-AG), the average weighted kappa = 0.70.
Ortega et al., 2013 19	Spain	Young adults (18-43 years)	Validity: 276 (72%) Reliability: 181 (73.48%)	Construct validity, Test-retest reliability (two weeks interval)	the 20-m shuttle run test	① the handgrip test (maximum handgrip strength), ② the standing broad jump test (SBJ)	no	the sit-and-reach test	① Reliability(weighted kappa coefficients): kappa statistic, ② Construct validity: ANCOVA (Bonferroni-adjusted pairwise comparison)	① Reliability: CRF, MF, SP-AG, F. Weighted Kappa ranged from 0.54 (MF) to 0.65 (overall fitness), the averaged Kappa = 0.59 ② Validity: higher self-rated fitness had higher objectively measured fitness.
Ortega et al., 2011 15	Nine European countries	Adolescents (12.5-17.5 years)	Reliability/validity: 277 (51%)	Construct validity, Test-retest reliability (two weeks interval)	the 20-m shuttle run test	① the handgrip test, ② the standing broad jump test (SBJ)	the 4x10-m shuttle run test	the back-saver sit-and-reach test	① Reliability(weighted kappa coefficients): kappa statistic, ② Construct validity: ANOVA and binary logistic regression	Perfect agreement in 65% (range 61%–70%) of the adolescents, perfect acceptable agreement in 97% (range 96–99) for the self-reported overall PF and main components, kappa coefficients for CRF, MF, SP–AG, F, and overall fitness were 0.58, 0.54, 0.60, 0.59 and 0.65.

Note: M = the average of physical fitness, n = the number of the 30-s chair stand test, cardiorespiratory fitness = CRF, muscular fitness = MF, speed and agility = SP-AG, flexibility = FL, EFA = exploratory factor analysis.

### 2.5 Data synthesis

All meta-analyses were performed using the Comprehensive Meta-Analysis Software (version 3), across specific age-groups with a minimum of three studies that reported reliability or validity coefficients for the IFIS in same age group. The random effects model was employed to compute the pooled effect size of reliability and validity of IFIS, which was deemed more suitable for heterogeneous data.^38^ Additionally, we computed the 95% confidence intervals (CI) for each pooled effect size. Cohen's weighted kappa coefficients were utilized to determine the pooled effect sizes, categorized as poor (0-0.20), fair (0.21-0.40), moderate (0.41-0.60), substantial (0.61-0.80), and perfect (0.81-1.0).^34^ Meanwhile, we assessed the heterogeneity of the average effect size using I^2^ statistic and Q statistics. We classified the I^2^ statistic into three levels: low heterogeneity (I^2^ < 25%), moderate heterogeneity (I^2^ < 50%), and high heterogeneity (I^2^ > 75%), respectively.^39^ Due to the limited number of studies included in each sub-population, we refrained from conducting moderator analyses. Moreover, a meta-analysis of construct validity was precluded by heterogeneity in the constructs assessed and the consistent lack of reported effect sizes.

## 3 Results

### 3.1 Study characteristics

**Fig. 1:**
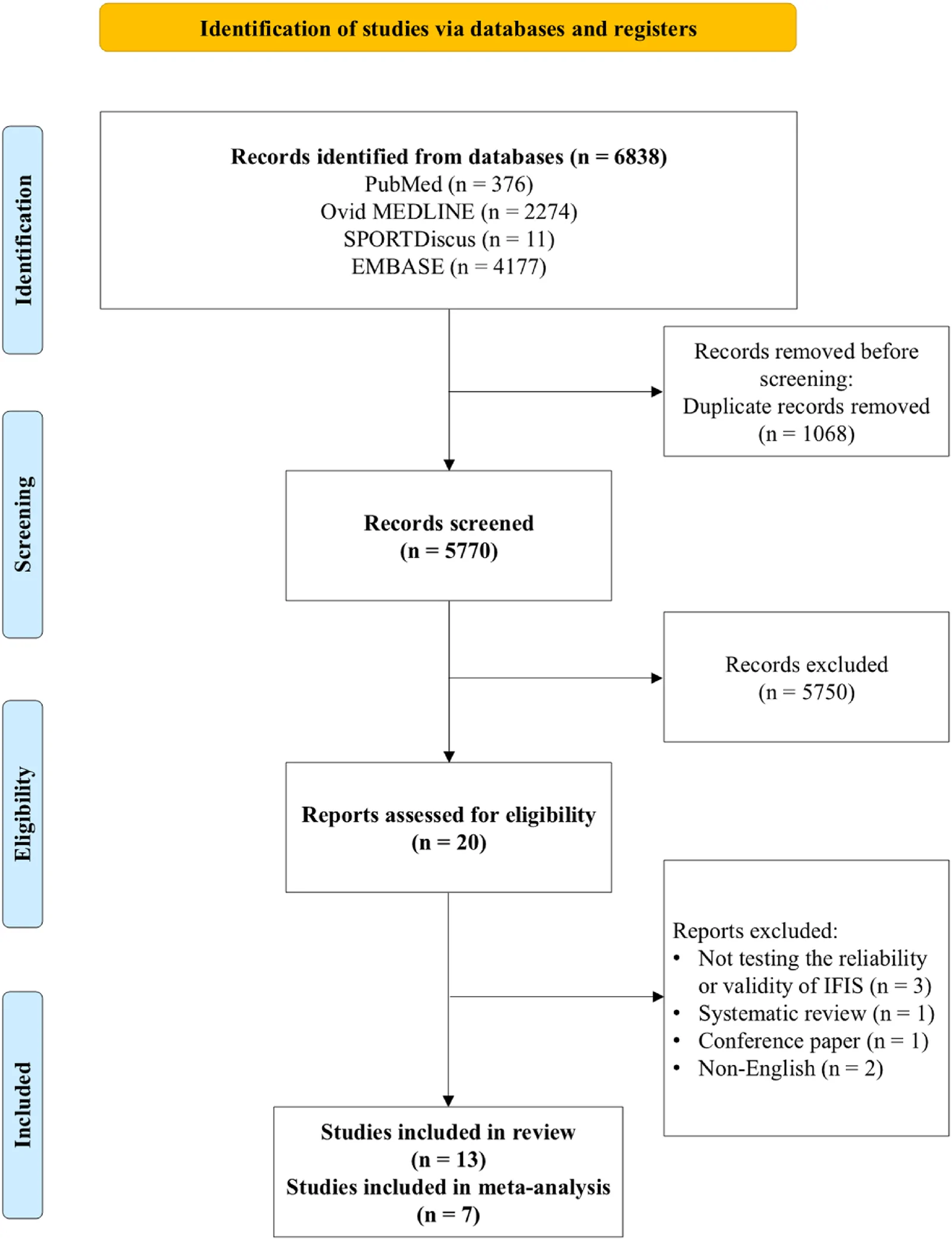
Fig. 1 Flow diagram of the study selection process.

A total of 6838 records were identified through database searches. After removing 1068 duplicates, 5770 records remained for title and abstract screening. Of these, 5750 were excluded as irrelevant. The full texts of 20 records were assessed for eligibility, with seven excluded for the following reasons: not testing the reliability or validity of IFIS (n = 3), being systematic reviews (n = 1), conference abstracts (n = 1), or non-English papers (n = 2). Finally, a total of 13 studies (9634 participants) met the inclusion criteria ^13^^,^^15^^,^^18^^,^^19^^,^^21^^,^^22^^,^^27^, ^28^, ^29^, ^30^^,^^35^, ^36^, ^37^ for analysis. The process of the literature search is depicted in Fig. 1.

Table 1 details the general information of the included studies. The included studies were conducted in Spain,^13^^,^^19^^,^^21^^,^^27^^,^^37^ China,^29^ Brazil,^30^^,^^35^ Colombia,^36^ Chile,^18^ France,^22^ and Sweden,^28^ respectively; and one study used data collected from nine European countries.^15^ Ten studies assessed the reliability of the IFIS, and sample size ranged from 101 to 3051. The validity of IFIS was examined in 12 studies, and the sample size ranged from 131 to 3051. The study populations represented a wide range of life stages. One study focused on preschool children under 5 years using proxy reports, whereas seven studies examined self-reported data from children and adolescents aged 5-18 years. Among adults, two studies involved pregnant women, one included women with fibromyalgia, and one investigated young adults aged 18-43 years. One study examined older adults over 65 years.

### 3.2 Methodological quality assessment

Table 2 shows the results of methodological quality of the included studies. There were eleven studies that were rated as high-quality in this review, and the remaining two studies were evaluated as moderate quality. Overall, the included studies were of a high methodological quality.

**Table 2:** Methodological quality assessment of included studies.

Reference	Reliability									Criterion Validity				Construct Validity					Quality level
	Item 1	Item 2	Item 3	Item 4	Item 5	Item 6	Item 7	Item 8	RoB	Item 1	Item 2	Item 3	RoB	Item 1	Item 2	Item 3	Item 4	RoB	
Débora de Almeida et al., 2025	A	G	A	/	G	G	G	G	No	/	/	/	/	/	/	G	G	No	High
Matelot et al., 2024	A	D	A	/	G	G	G	G	No	/	/	/	/	G	G	G	G	No	High
Sánchez-López et al., 2023	A	G	A	/	G	G	G	G	No	/	G	G	No	G	G	G	G	No	High
Bao et al., 2022	A	G	G	/	G	G	A	G	No	/	/	/	/	/	/	/	/	/	High
Henström et al., 2021	/	/	/	/	/	/	/	/	/	/	G	G	No	/	/	/	/	/	High
Romero-Gallardo 2020	/	/	/	/	/	/	/	/	/	/	/	/	/	G	G	G	G	No	High
De Moraes 2019	A	G	A	/	G	D	G	G	No	/	G	G	No	G	G	G	G	No	High
Ramírez-Vélez 2017	A	D	D	/	G	G	A	G	Serious	/	/	/	/	G	G	G	G	No	Moderate
Merellano-Navarro 2017	/	/	/	/	/	/	/	/	/	/	/	/	/	G	G	G	G	No	High
Álvarez-Gallardo 2016	A	D	D	/	G	G	G	G	Serious	/	/	/	/	G	G	G	G	No	Moderate
Sánchez-López 2015	A	G	D	/	G	G	A	G	No	/	/	/	/	G	G	G	G	No	High
Ortega 2013	A	G	D	/	G	G	A	G	No	/	/	/	/	G	G	G	G	No	High
Ortega 2011	A	G	D	/	G	G	G	G	No	/	/	/	/	G	G	G	G	No	High

Note: overall scores = total scores of all items of reliability/validity, “/” = not applicable or not studied. I = inadequate, D = doubtful, A = adequate, G = very good; RoB = risk of bias.

Test-retest reliability.

Item 1 = Were patients stable in the interim period on the construct to be measured?.

Item 2 = Was the time interval appropriate?.

Item 3 = Were the test conditions similar for the measurements (e.g., type of administration, environment, instructions)?.

Item 4 = For continuous scores: Was an intraclass correlation coefficient (ICC) calculated?.

Item 5 = For dichotomous/nominal/ordinal scores: Was kappa calculated?.

Item 6 = For ordinal scores: Was a weighted kappa calculated?.

Item 7 = For ordinal scores: Was the weighting scheme described (e.g., linear, quadratic)?.

Item 8 = Were there any other important flaws in the design or statistical methods of the study?.

Criterion validity.

Item 1 = Were correlations, or the area under the receiver operating curve calculated (for continuous scores)?.

Item 2 = For dichotomous scores: Were sensitivity and specificity determined?.

Item 3 = Were there any other important flaws in the design or statistical methods of the study?.

Construct validity.

Item 1 = Is it clear what the comparator instrument(s) measure(s)?.

Item 2 = Were the measurement properties of the comparator instrument(s) sufficient?.

Item 3 = Was the statistical method appropriate for the hypotheses to be tested?.

Item 4 = Were there any other important flaws in the design or statistical methods of the study?.

### 3.3 Reliability

Across the included studies, test-retest reliability was evaluated in Brazilian children and adolescents,^30^^,^^35^ Chinese children and adolescents,^29^ Colombian youths,^36^ European adolescents (the combined sample),^15^ French children,^22^ Spanish populations including preschool children,^21^ children (9-12 years),^37^ young adults^19^ and women with Fibromyalgia.^13^ Concerning test-retest interval, six studies^15^^,^^19^^,^^21^^,^^29^^,^^37^ completed the IFIS on two occasions with a 14-day period whilst the other studies used a one-week interval.^13^^,^^22^^,^^35^^,^^36^ As shown in Table 3, the included studies reported a moderate to almost perfect reliability of the IFIS.

**Table 3:** Measurement properties of reliability and validity of the included studies in this review.

Author/year	Reliability						Validity					
	Overall	CRF	MF	SP-AG	FL	Balance	Overall	CRF	MF	SP-AG	FL	Balance
Débora de Almeida et al., 2025	S	S	S	S	G	/	/	/	/	/	/	/
Matelot et al., 2024	S	S	S	M	S	/	/	/	/	/	/	/
Sánchez-López et al., 2023	M	G	M	M	/	M	/	P	P	P	/	P
Bao et al., 2022	M	M	M	M	M	/	/	/	/	/	/	/
Henström et al., 2021	/	/	/	/	/	/	/	/	/	/	/	/
Romero-Gallardo 2020	/	/	/	/	/	/	?	?	?	/	?	/
De Moraes 2019	G	G	G	G	G	/	C: M	C: S	C: M	C: M	C: F	/
							A: M	A: F	A: M	A: M	A: M	
Ramírez-Vélez 2017	G	G	G	G	G	/	?	?	?	?	?	/
Merellano-Navarro 2017	/	/	/	/	/	/	?	?	?	?	?	/
Álvarez-Gallardo 2016	M	M	M	M	M	/	?	?	?	?	?	/
Sánchez-López 2015	S	S	S	S	S	/	?	?	?	?	?	/
Ortega 2013	S	M	M	M	M	/	?	?	?	?	?	/
Ortega 2011	S	M	M	M	M	/	?	?	?	?	?	/

Note: C = children, A = adolescents; “G” = almost perfect (0.81–1.0), “S” = substantial (0.61–0.80), “M” = moderate (0.41–0.60), “F” = fair (0.21–0.40) 34, “P” = poor (0-0.20); “?” = weighted Kappa was not reported (indeterminate); “/” = not available.

**Table 4:** Pooled estimated weighted kappa of reliability and validity of the components of the IFIS.

Reliability	Items	No. of studies (weighted kappa)	Model	Weighted kappa (95%CI)	p value	Heterogeneity			
						Q value	df (Q)	*I*^2^	*Tau*^2^
	Overall fitness	7 (12)	Fixed	0.647 (0.629-0.664)	<0.001	899.283	11	98.777	0.237
			Random	0.750 (0.600-0.848)	<0.001				
	CRF	7 (12)	Fixed	0.638 (0.620-0.656)	<0.001	816.758	11	98.653	0.215
			Random	0.752 (0.612-0.846)	<0.001				
	MF	7 (12)	Fixed	0.659 (0.641-0.675)	<0.001	350.584	11	96.862	0.091
			Random	0.724 (0.630-0.798)	<0.001				
	SP-AG	7 (12)	Fixed	0.669 (0.652-0.685)	<0.001	655.508	11	98.322	0.173
			Random	0.764 (0.645-0.847)	<0.001				
	FL	7 (12)	Fixed	0.641 (0.623-0.659)	<0.001	335.094	11	96.717	0.087
			Random	0.725 (0.633-0.796)	<0.001				

CRF: cardiorespiratory fitness; MF: muscular fitness; SP-AG: speed-agility; FL: flexibility.

**Fig. 2:**
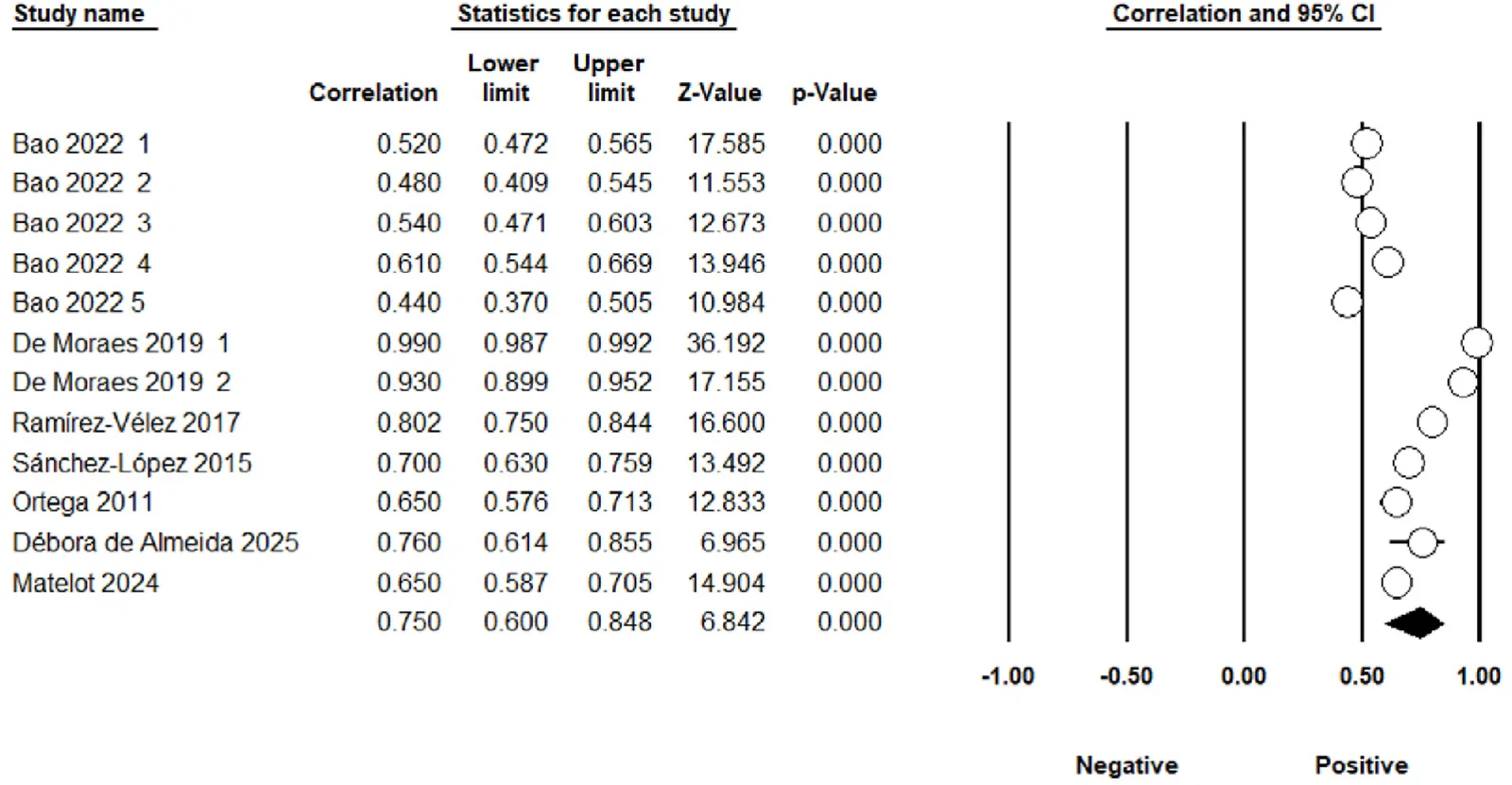
Forest plot on the reliability of overall fitness in children and adolescents.

**Fig. 3:**
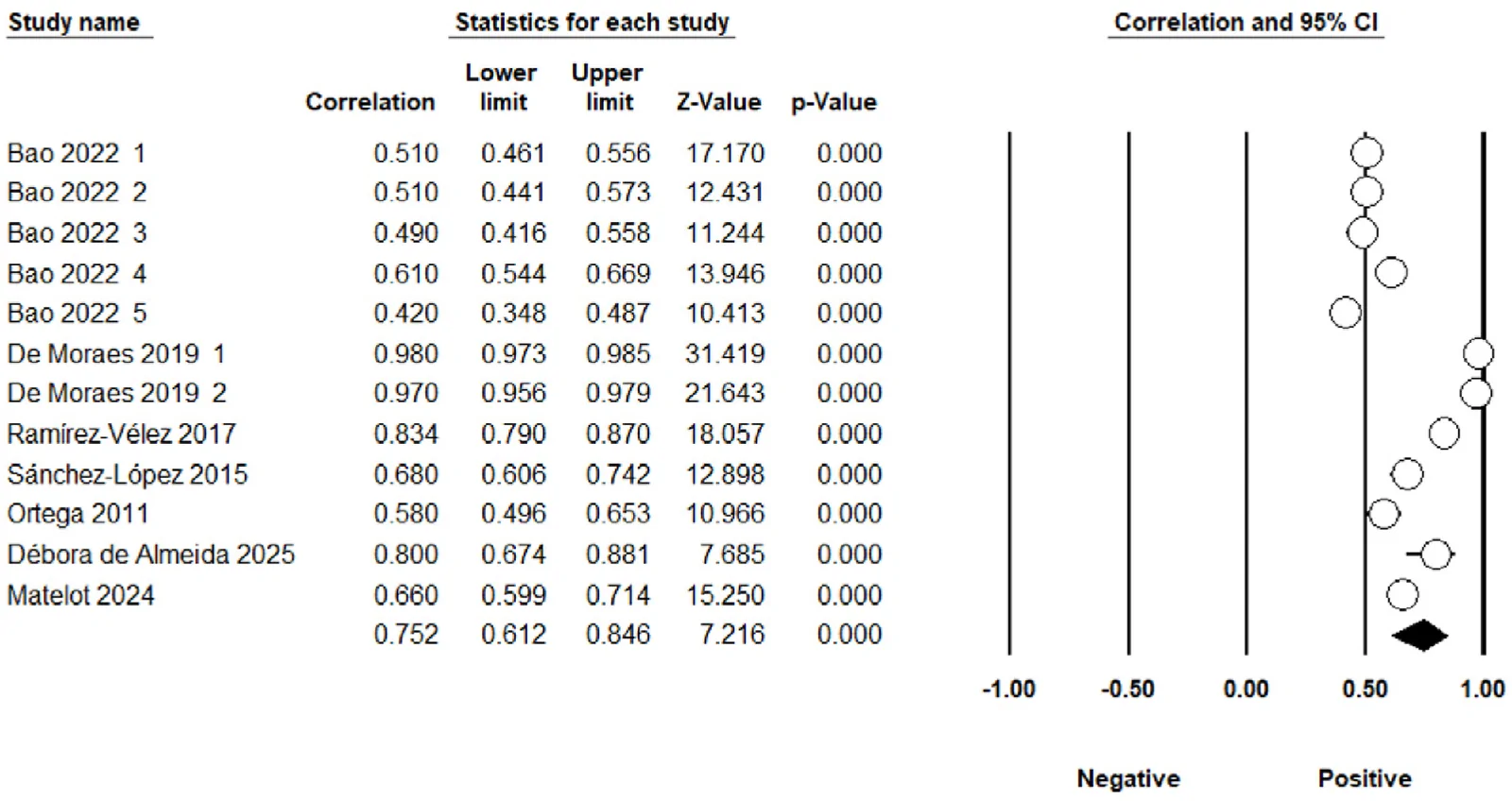
Forest plot on the reliability of cardiorespiratory fitness in children and adolescents.

**Fig. 4:**
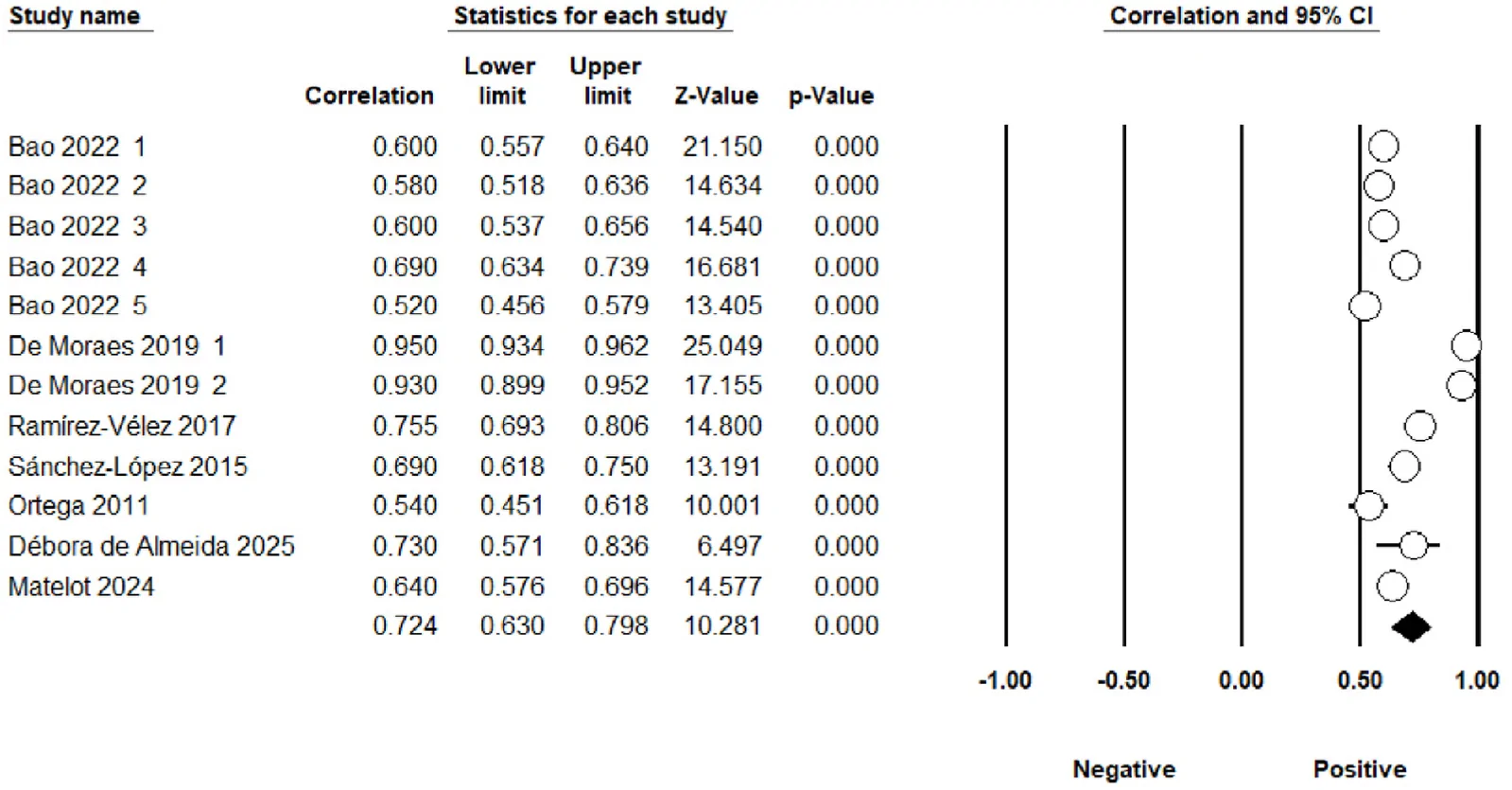
Forest plot on the reliability of muscular fitness in children and adolescents.

**Fig. 5:**
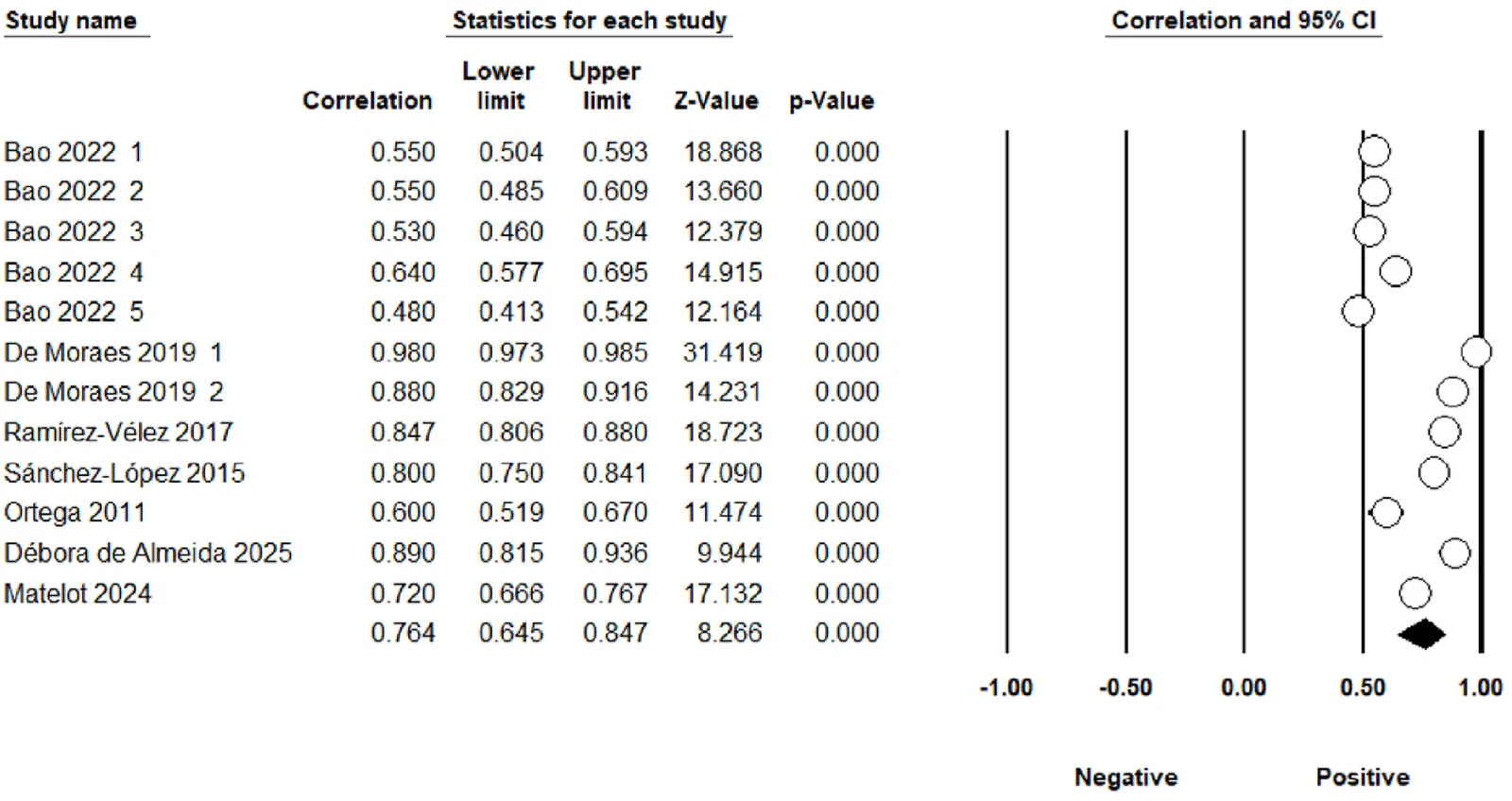
Forest plot on the reliability of speed-agility in children and adolescents.

**Fig. 6:**
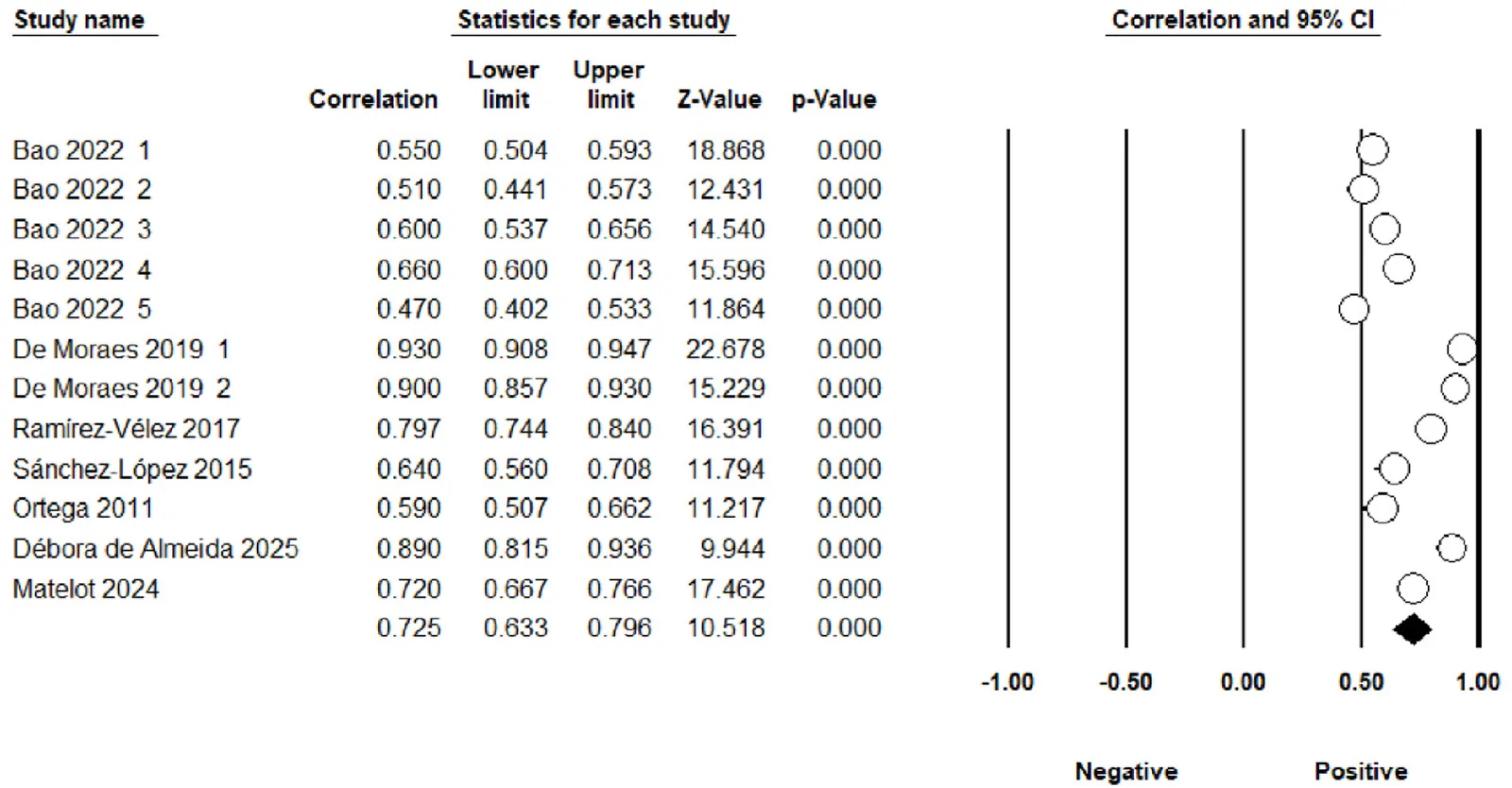
Forest plot on the reliability of flexibility in children and adolescents.

Table 4 presents the results of the meta-analysis for reliability in children and adolescents (Fig. 2, Fig. 3, Fig. 4, Fig. 5, Fig. 6)0.^15^^,^^22^^,^^29^^,^^30^^,^^35^, ^36^, ^37^ We found that the IFIS is a reliable instrument to assess the components of fitness. Specifically, a substantial test-retest reliability was observed for overall fitness (pooled weighted kappa = 0.750, 95%CI = 0.600-0.848, Q = 899.283, I^2^ = 98.777), CRF (pooled weighted kappa = 0.752, 95%CI = 0.612-0.846, Q = 816.758, I^2^ = 98.653%), MF (pooled weighted kappa = 0.724, 95%CI = 0.630-0.798, Q = 350.584, I^2^ = 96.862%), SP-AG (pooled weighted kappa = 0.764, 95%CI = 0.645-0.847, Q = 655.508, I^2^ = 98.322%), and FL (pooled weighted kappa = 0.725, 95%CI = 0.633-0.796, *Q* = 335.094, *I*^*2*^ = 96.717%). The differences between studies contributed to heterogeneity across the analyzed components of the IFIS. Furthermore, two studies focusing on adults were excluded from the meta-analysis as they only included women.^13^^,^^19^ In a study of 181 young adults, Ortega et al. evaluated the reliability of IFIS, reporting moderate agreement, with an average weighted Kappa of 0.59.^19^ Álvarez-Gallardo et al. evaluated a total of 101 women diagnosed with Fibromyalgia, and also reported moderate agreement with an average weighted Kappa of 0.45.^13^

### 3.4 Validity

Validity was evaluated in Brazilian children and adolescents,^30^^,^^35^ Colombian youths,^36^ Chilean older adults,^18^ European countries,^15^ French children,^22^ Spanish populations including preschool children,^21^ children (9-12 years),^37^ young adults,^19^ women with Fibromyalgia^13^ and pregnant women,^27^ as well as Swedish pregnant women.^28^

When examining the validity of CRF within the IFIS against field-based measures, the 20-m shuttle run test was used in seven studies,^15^^,^^19^^,^^21^^,^^22^^,^^35^, ^36^, ^37^ the 6-min walk test was used in three studies,^13^^,^^18^^,^^28^ and the Bruce test was used in one study,^27^ respectively. Regarding MF assessment, the handgrip test emerged as a popular choice for evaluating upper body strength, utilized in nine studies.^15^^,^^18^^,^^19^^,^^21^^,^^27^^,^^28^^,^^35^, ^36^, ^37^ Additionally, the arm curl test was each applied in a single study.^13^ For lower body MF, the standing broad jump test was used in seven studies,^15^^,^^19^^,^^21^^,^^22^^,^^35^, ^36^, ^37^ while the 30-s chair stand was conducted in two studies.^13^^,^^18^ This highlights the dominance of handgrip test for upper body evaluation. The 4 × 10-m shuttle run test was used to test the SP-AG in five studies; ^15^^,^^21^^,^^35^, ^36^, ^37^ the timed up and go test,^18^ running as fast as possible in 5s^22^ and the 8-foot up-and-go test^13^ were used in one single study, respectively. For FL, the sit-and-reach test was frequently used to assess validity in seven studies,^13^^,^^15^^,^^18^^,^^19^^,^^35^, ^36^, ^37^ and the back-scratch test was used in two studies,^18^^,^^27^ the stand-and-reach test in one study.^22^ Owing to the potential risk for pregnant women, SP-AG was not tested in two studies.

Due to methodological heterogeneity and insufficiently reported data for pooling, validity results were presented as a narrative rather than a quantitative synthesis (Table 3). According to the Landis and Koch^34^ benchmarks, moderate construct validity was reported in children and adolescents,^35^ but poor construct validity in preschool children.^21^ Further, 12 included studies assessed the construct validity of the IFIS, reporting that participants who self-rated higher fitness had higher objectively measured fitness.^13^^,^^15^^,^^18^^,^^19^^,^^21^^,^^22^^,^^27^^,^^28^^,^^30^^,^^35^, ^36^, ^37^.

## 4 Discussion

The IFIS is a self-rated instrument to assess individual's PF level with relatively low testing burden and cost, which has been used widely in many settings. In this study, we identified 13 studies, published from 2011 to 2025, that adequately evaluated the IFIS for reliability and/or validity. The findings of this review demonstrate that the IFIS provides sufficient reliability in children and/or adolescents with substantial pooled Kappa values from 0.724 to 0.764, being the reliability slightly lower for muscular strength and flexibility than for cardiorespiratory fitness, speed-agility and overall fitness. In terms of validity, acceptable validity was reported across studies. However, owing to the heterogeneity in methodology, this review was not able to synthesize the effect size of validity.

### 4.1 Reliability of the IFIS

A previous meta-analysis pooled Kappa estimates across heterogeneous populations (children, adolescents, adults, women with fibromyalgia),^26^ likely resulting in the high heterogeneity between studies. To address this limitation, we conducted an updated meta-analysis and pooled weighted kappa values only for the same age group. The pooled findings from seven studies indicate substantial test-retest reliability across all IFIS domains in children and adolescents, supporting its use as a self-report measure of PF in these populations. However, evidence remains scarce in preschool children, adults and clinical populations, highlighting the need for further psychometric evaluation in these groups.

Nevertheless, some variability was observed in children and adolescents across studies and cultural settings. In general, reliability estimates tended to be higher in South American and Asian samples. Specifically, excellent kappa coefficients were reported in Brazilian^35^ and Colombian children and adolescents,^36^ whilst moderate kappa coefficients were reported in European adolescents,^15^ Spanish children,^37^ Chinese children and adolescents.^29^ Concerning the differences across these studies, one possible explanation is that the IFIS may have been administered in different language version and within populations differing in age, educational background, and cognitive maturity. As a self-report instrument, its reliability may also be influenced by translation quality, cultural adaptation, and differences in how respondents interpret the response categories. Therefore, future studies should evaluate the reliability of the IFIS across cultural backgrounds.

Despite promising findings in children and adolescents, evidence regarding the psychometric properties of the IFIS remains limited in preschool children, older adults, and the clinical population. Our findings indicated a moderate test-retest reliability of the IFIS in young adults^19^ and in women with fibromyalgia.^13^ However, lower test-retest reliability were reported in women with fibromyalgia.^13^ This may be attributed to the health condition of the participants, which was found to be affected by fatigue levels and the severity of symptoms.^13^ Indeed, biological variability and instrument variability may be potential problems in the measurement of reliability.^13^ For broader public health application, further studies are needed to assess the reliability in clinical and other special populations.

Methodological factors may partially explain the variability in reliability estimated across studies. In particular, studies employing shorter test-retest intervals generally reported higher reliability coefficients,^35^^,^^36^ suggesting that the length of the assessment interval may influence reliability estimates. Despite the simple question and construct of the IFIS, shorter intervals may increase recall effects and inflate reliability estimates. In contrast, multi-domain questionnaires may introduce cognitive overload, potentially increase measurement error and reduce response stability.^40^ Nevertheless, the two-week interval recommended by the COSMIN guidelines remains the preferred approach for future reliability studies.^32^ Therefore, there is a need to discern whether the time interval between test administration influences the reliability coefficients of the IFIS. Furthermore, the developmental challenges associated with self-report assessment in preschoolers may affect the reliability and validity of the IFIS in this population.^35^ Future research should examine the psychometric properties of parents and/or guardians' proxy reported version of the IFIS. However, the veracity of this claim must be empirically tested, as parents and guardians may overestimate children's physical fitness.

### 4.2 Validity of the IFIS

Most included studies evaluated the construct validity. According to the conclusions of these included studies, the construct validity of the IFIS was acceptable. However, due to a lack of information regarding validity coefficients, validity results of included studies in this review were equivocal. To facilitate comparison between studies, quantified validity coefficients are encouraged to be reported in future validity studies. Furthermore, due to the few validity studies of each population subgroup, more validity studies are encouraged to evaluate the feasibility of IFIS in other countries or regions, as well as adults, older adults, and clinical populations.

Given that higher self-perception of PF in children and adolescents yielded greater confidence in self-assessment, a majority of children^37^ and adolescents^15^ reported “very good” physical fitness. In contrast, women and older adults tended to select central response categories when rating their physical fitness,^13^^,^^18^^,^^27^ whilst over-reporting was found in males (except for flexibility).^37^ To avoid low levels of ceiling effect (i.e. most of the participants reported “very good” physical fitness) and floor effect (i.e. most of the participants reported “very poor” physical fitness),^18^ four studies re-classified the categories.^13^^,^^18^^,^^19^^,^^27^ Therefore, to avoid the overestimate of self-reported PF, accurate measures (i.e., provide interpretation and instruction for participants) need to be conducted in future self-reported research.

In this review, two studies evaluated the construct validity of the IFIS only in women, including pregnant women^27^ and women with fibromyalgia.^13^ Romero-Gallardo et al. revealed that the IFIS can provide relevant information about the level of PF, as well as discriminating the level of health-related quality of life of pregnant women.^27^ In the study of Álvarez-Gallardo et al., however, no significant differences were found between “very poor” and “good/very good” fitness categories, which was attributed to only a few participants reporting good/very good fitness; thus, the IFIS may be of limited utility in women with high levels of fitness in clinical research.^13^ Therefore, more validity studies should be encouraged to identify the feasibility of IFIS in clinical or other specific populations.

### 4.3 Methodological quality assessment

The quality of the included studies was evaluated using the COSMIN checklist,^32^ and over 100 participants were considered to be a sufficient sample size. In this review, the sample size of the included studies was sufficient, as all the included studies resulted in a sample size greater than 100. Besides, the interval time of two reliability studies was one week, and a score of “doubtful” was assigned for Item 2 in reliability studies. Although two-week is generally considered an appropriate time interval in the COSMIN checklist, the included studies suggest that the shorter time interval had little effect on the reliability of the IFIS. However, respondents may remember the answers that they gave in the first occasion with a shorter time interval, which may lead to the overestimation of reliability.^41^ Therefore, careful consideration should be taken into the study design of test-retest reliability studies. Moreover, all the included studies evaluated construct validity, but no information about kappa or correlation coefficients was reported. Therefore, it is difficult to explain any heterogeneity in the findings through quantitative values. It is strongly recommended to conduct the validation study and report the results by adhering to the COSMIN Checklist.^42^

### 4.4 Strengths and limitations

Several strengths of the current study are worthwhile to acknowledge. First, we updated the evidence synthesizing the available evidence on the validity and reliability of the IFIS. Second, we used the COSMIN checklist to assess the methodological quality of the included studies in this review, which is specifically designed to improve the credibility of the selected health-related instruments. Despite the aforementioned strengths, a few study limitations have to be considered in light of understanding the study findings. Although we sought to include the maximal number of studies, non-English journal papers were excluded from this review, which may be a potential selection bias. Additionally, we recommend that the study design of the original studies follow COSMIN guidelines. Due to the relatively small number of studies identified examining the measurement properties of IFIS, it is impossible to identify feasibility and acceptability of the IFIS in population subgroups through this review study.

## 5 Conclusion

This review supports that the IFIS has acceptable test-retest reliability in children and adolescents, supporting its use for population surveillance. However, evidence in preschooler, adults and clinical populations remains limited, warranting further reliability studies. Construct validity of the IFIS was reported acceptable across studies, however, to enable a quantitative synthesis of the validity evidence, future studies should standardize reporting. As existing research is restricted to European or South America, cross-cultural evaluation is needed to establish reliability and validity across diverse regions and population subgroups, using rigorous translation and cultural adaptation procedures and, where feasible, measurement invariance testing.

## Author contributions

Conceptualization: RB., S.C. Literature search: R.B. Abstract screening, full-text eligibility assessment, data extraction, and critical appraisal: R.B., S.C. Data synthesis: R.B., S.C., Y.Z. Original draft of manuscript: R.B., S.C. Manuscript review and edit: R.B., S.C., Y.Z., F.B.O., C.D., K.Z. All authors read and approved the final manuscript.

## Data availability statement

Data sharing is not applicable to this article as no new datasets were generated or analyzed during this study.

## Conflicts of interest

The authors declare no conflicts of interest.
